# Emerging biomarkers for the detection of cardiovascular diseases

**DOI:** 10.1186/s43044-022-00317-2

**Published:** 2022-10-20

**Authors:** Sreenu Thupakula, Shiva Shankar Reddy Nimmala, Haritha Ravula, Sudhakar Chekuri, Raju Padiya

**Affiliations:** 1 Department of Biochemistry, Osmania University, Amberpet, Hyderabad, Telangana 500007 India; 2 Department of Plant Sciences, University of Hyderabad, Gopanpalle, Hyderabad, Telangana 500019 India; 3 Department of Genetics, Osmania University, Amberpet, Hyderabad, Telangana 500007 India

**Keywords:** Biomarkers, Biomolecular systems, Cardiovascular disease, Cardiac markers, Diagnosis, Therapeutics

## Abstract

**Background:**

The prevalence of cardiovascular disease (CVD) has been continuously increasing, and this trend is projected to continue. CVD is rapidly becoming a significant public health issue. Every year there is a spike in hospital cases of CVD, a critical health concern in lower- and middle-income countries. Based on identification of novel biomarkers, it would be necessary to study and evaluate the diagnostic requirements or CVD to expedite early detection.

**Main body:**

The literature review was written using a wide range of sources, such as well-known medical journals, electronic databases, manuscripts, texts, and other writings from the university library. After that, we analysed the specific markers of CVD and compiled a systematic review. A growing body of clinical research aims to identify people who are at risk for cardiovascular disease by looking for biomolecules. A small number of biomarkers have been shown to be useful and reliable in medicine. Biomarkers can be used for a variety of clinical applications, such as predicting heart disease risk, diagnosing disease, or predicting outcomes. As a result of the ability for a single molecule to act as a biomarker, its usefulness in medicine is expected to increase significantly.

**Conclusions:**

Based on assessing the current trends in the application of CVD markers, we discussed and described the requirements for the application of CVD biomarkers in coronary heart disease, cerebrovascular disease, rheumatic heart disease, and other cardiovascular illnesses. Furthermore, the current review focuses on biomarkers for CVD and the procedures that should be considered to establish the comprehensive nature of the expression of biomarkers for cardiovascular illness.

## Background

Coronary heart disease (CAD), cerebrovascular disease (CD), rheumatic heart disease (RHD), and other cardiovascular illnesses are non-communicable diseases and leading causes of global mortality [1]. CVD produces more fatalities yearly than any other disease, accounting for 17.9 million deaths per annum, which is about 31% of all worldwide deaths. Coronary heart disease claimed the lives of 7.3 million people, while stroke claimed the lives of 6.2 million [2]. By 2030, 23.6 million individuals will have died from CVD, principally heart disease and stroke. Biomarkers are used to diagnose and monitor cardiovascular disease to reduce the mortality rate [3].

**Fig. 1:**
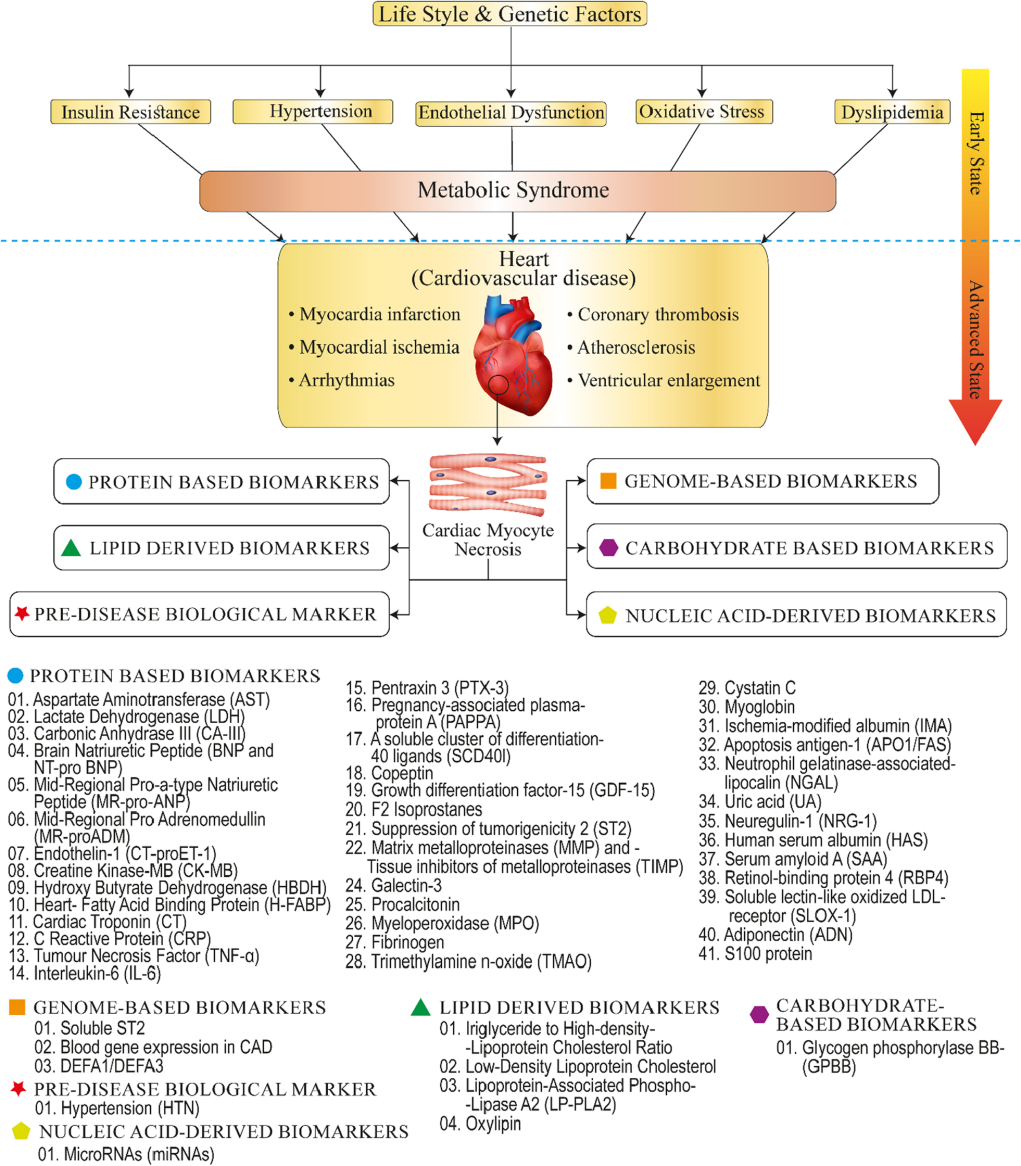
Schematic illustration of emerging biomarkers for the cardiovascular diseases respective to the biomolecular information currently available in the literature

Cardiac biomarkers are bio-molecular components obtained through venal punctures and secreted in the blood during an injured or strained heart [4]. Heart biomarker tests may assist in evaluating a person's risk of cardiac illnesses and monitoring and treating someone suspected of having an acute coronary syndrome (ACS) and cardiac ischemia. Biomarkers are a powerful tool for identifying high-risk people, promptly and reliably diagnosing illness conditions, and effectively diagnosing and treating patients [5, 6]. Most other biomarkers seem to be up-regulated or down-regulated in illnesses other than CVD. Current trends in identifying the number of biomarkers that may consistently aid prognosis in the early stages of the disease are limited. This review provides an overview of commonly used CVD biomarkers based on the carbon skeleton from which they are formed. Some of their merits and drawbacks, proposing a method for future, innovative biomarker research are shown in Fig. 1 and Table 1.

**Table 1:** Notable emergent biomarkers in cardiovascular diseases

Name of Marker	Derivative of	Source of tissue	Cause of increase	Time point to increase	Time point to back normal	Diagnostic use
Cardiac troponin	Regulatory protein complex. T and I cardiac-specific isoforms	Heart	Heart injury	3–4 h after injury	7–10 days	Myocardial infarction and degree of damage
C reactive protein	Protein derived. Acute phase protein	Liver	Raise in Inflammation	4–6 h after inflammation	Decrease (36–50 h.) after resolution of inflammation	Chronic inflammatory disease and risk of heart disease
Myeloperoxidase	Protein derived	azurophilic granules of polymorphonuclear neutrophils and macrophages	Coronary artery disease and Inflammation	2 h after onset of symptoms	–	Diagnosis and risk stratification of acute coronary artery syndrome
CK-MB	Myocardium-related isoenzymes of CK	Heart and Skeletal muscles	When injury to heart and skeletal muscle cells	4to 6 h after heart muscles injury, peak level at 24 h	Within 48–72 h, it will return to normal	Less than Troponin in specificity. Used to diagnostic re-infarction
Ischemia modified albumin (IMA)	Protein derived	Heart tissue	when circulating serum albumin contacts ischemic heart tissues	Rise within minutes of transient ischemia. Peak within 6 h	–	It is a good discriminator between ischemic and non-ischemic patients
Myoglobin	Protein derived, oxygen sorting protein	Hearty and skeletal muscles	When injury to heart and skeletal muscle cells	After injury 2–4 h, peak level at 8 to 12 h	Return to normal within 24 h	Early diagnosis in addition to Troponin
Fibrinogen	Glycoprotein	Liver	Tissue and vascular injury, it may elevate in inflammation	5 days after injury	–	Bleeding disorders and risk of cardiovascular diseases
TMAO	Amino acid derivative	Intestinal lumen	Cardiovascular and Renal disease	Increase within 24 h	–	To predict heart attack, stroke and kidney disease
Cystatin- C	Protein derivative	All nucleated cells	Acute kidney injury and cardiovascular disease	When acute kidney injury develops, after 8 h of cardiac surgery	–	Early detection of acute kidney injury in cardiac surgery patients
Lipoprotein-associated phospho lipase A2	Lipid derivative	Leukocytes	Cardiovascular inflammation and heart failure	After 24 h of inflammation	–	To help to determine risk of CVD’s and coronary heart disease and ischemic stroke
Oxylipins	Lipid derivative	All nucleated cells	Increased in cardiovascular diseases			Develop early diagnostic tools for CVD’s

## Main text

### Carbohydrate-based biomarkers

#### Glycogen phosphorylase BB (GPBB)

The heart is highly susceptible to CO (carbon monoxide)-induced hypoxia due to its high oxygen demand. Due to a lack of apparent symptoms or specific electrocardiogram (ECG) changes, CO poisoning in cardiovascular patients may go undiagnosed and untreated. The importance of early and particular markers for myocardial hypoxia is growing. One candidate is the glycogen phosphorylase BB isoenzyme (GPBB) [7–9]. GPBB is a novel cardiac marker that might help identify myocardial ischemia early [10–12]. In hypoglycaemia or hypoxic settings, GPBB is an important enzyme that catalyses the first rate-limiting step in the degradation of glycogen to glucose 1-phosphate [13, 14].

In humans, three distinct isoforms have been identified: GPMM (present in muscles), GPLI (liver), and GPBB (brain). The diagnostic sensitivity of myoglobin and ischemia-modified albumin (IMA) is good. However, there is no heart muscle specificity [15]. GPBB is a more sensitive marker for diagnosing ACS within 4 h after the beginning of chest pain [16, 17], with a sensitivity of 81% and a specificity of 93%. This research revealed that GPBB is an excellent clinical marker of ischemia and may be used to diagnose ACS [15]. Although there is an expected increase in plasma GPBB concentration during pregnancy, which is also seen in the brain, there is no absolute specificity for identifying the cardiac injury. GPBB in plasma is a surrogate marker for acute coronary syndromes in many investigations [11, 16, 18]. As a result, GPBB may be employed as a secondary biomarker for detecting acute myocardial infarction [12].

### Protein-based biomarkers

#### Aspartate aminotransferase (AST)

Aspartate aminotransferases, also called liver transaminases, are intracellular enzymes catalysing the transamination reaction between amino acids and ketonic acid [19]. Because it is present mainly in hepatocytes, AST is a commonly used serum marker of liver disease. Recent research has shown that liver transaminases can independently predict cardiac-related morbidity and mortality. The first cardiac biomarker, AST, was discovered in the liver, heart, skeletal muscle, brain, and kidney in 1954. It does not help diagnose AMI due to the lack of specificity of cardiac tissue [20, 21].

Total CK levels were tested for AMI in 1959 because it was an excellent indicator of skeletal muscle damage[22]. In 1960, LDH was used to diagnose AMI [23]. Finally, in 1979, the World Health Organization (WHO) approved a panel of CK, AST, and LDH to diagnose AMI [24]. 

#### Lactate dehydrogenase (LDH)

The intracellular enzyme lactate dehydrogenase (LDH), which is widely distributed, catalyses the conversion of pyruvic acid, the final by-product of the glycolytic process, to lactate. Since the 1960s, LDH has been employed to diagnose acute myocardial infarction as a marker of heart dysfunction. LDH is composed of two self-contained subunits, resulting in the formation of five isozymes. Each isozyme is expressed in a distinct organ: LDH_1_ is expressed in cardiomyocytes, LDH_3_ is found in lung tissue, and LDH_5_ is expressed in hepatocytes [25, 26]. Many organs, including skeletal muscle, kidney, liver, heart, lung, and erythrocytes, express LDH. LDH contains five isoenzymes, one of which is found in nature. Although it has been reported that an LDH_1_-to-LDH_2_ ratio higher than 1 is specific for AMI, it is presently not employed in diagnosing AMI [27]. LDH is now only used to differentiate acute from subacute myocardial infarction (MI) in patients with positive troponins whose CK and CK-MB readings have reverted to normal levels when they arrive at the hospital late in the disease [28].

#### Carbonic anhydrase III (CA-III)

Red blood cell primary protein constituents, carbonic anhydrases (CAs), are zinc-containing metalloproteinases that accelerate the hydration and dehydration of carbon dioxide [29]. CAs are found in various tissues and participate in multiple physiological functions, including gluconeogenesis, acid–base homeostasis, adipogenesis, and calcification [30]. CA-III protein is a unique member of the CAs family; it has a role in various disorders, including type-2 diabetes, MI, and skeletal muscle injury. CA-III protein is a cytosolic enzyme with lower activity than carbon dioxide hydratase [31]. It has been shown to scavenge oxygen radicals *in vivo* and increase survival from oxidative injury. The cytosolic isoform CA-III is abundant in skeletal muscles [32]. This enzyme possesses antioxidative properties and is critical for maintaining intracellular pH homeostasis. Given that high blood CA-III levels, often in conjunction with other muscle-specific proteins, are commonly used to diagnose overall muscle injury, it seemed worthwhile to review current CA-III investigations [33]. 

#### Brain natriuretic peptide (BNP and NT-pro BNP)

As a result of myocardial stretching, BNP and 32 amino acid (-a.a) N-terminal (NT)-proBNP are continually released from the ventricles into the bloodstream. The prohormone brain natriuretic peptide (proBNP), a 108-a.a peptide cleaved into the 76-a.a. BNP and 32-a.a. NT-proBNP, is produced as a result [34]. A sixfold increase in half-life compared to that of BNP is seen, and the peptide is eliminated from the body by many processes, including those involving the renal system and other systems [35]. BNP and NT-proBNP are high in individuals with heart failure, making them valuable diagnostic indicators for the disease. Brain natriuretic peptide (BNP) regulates several physiological and pathological processes [36]. BNP has been demonstrated to be involved in natriuresis, diuresis, vasodilatation, and inhibition of the sympathetic nervous system in addition to the renin–angiotensin–aldosterone system [37, 38]. Studies have shown that the function of BNP also influences diseases associated with the pathways mentioned above to a certain extent [39]. 

#### Mid-regional pro-a-type natriuretic peptide (MR-pro-ANP)

Natriuretic peptides are the gold standard in heart failure biomarkers and have been thoroughly researched in many clinical settings [40, 41]. In particular, B-type natriuretic peptide (BNP) is generated from scratch in response to ventricular stretch brought on by pressure and volume overload in both healthy adults and patients with left ventricular (LV) dysfunction [42]. N-terminal pro-B-type natriuretic peptide (NT-proBNP) is an essential cardiac biomarker that correlates well with left ventricular end-diastolic pressure and wall pressure. It has high sensitivity and specificity for the differential diagnosis of HF in patients with acute dyspnoea and has significant value in the diagnosis, evaluation, and prognosis of patients with either acute or chronic HF. Recently, a mid-regional sequence of pro-a-type natriuretic peptide (MR-pro-ANP), which is a more stable intermediate of the natriuretic peptides, was effectively employed in the clinical as a biomarker of prognosis and diagnosis of acute HF [18, 43]. 

#### Mid-regional pro-adrenomedullin (MR-proADM)

Adrenomedullin (ADM) is a vasoactive peptide in a human pheochromocytoma3 tumour. Even though a few organs and tissues produce ADM, it is principally synthesized by vascular endothelial cells and has a wide range of physiological functions [44, 45]. Patients with hypertension, congestive heart failure or myocardial infarction, renal disorders (including kidney failure and diabetes mellitus), acute phase of stroke (including septic shock), and arterial stiffness have been observed to have higher plasma ADM levels (including arterial stiffness). The magnitude of the increase is proportional to the severity of the vascular damage [46]. Sepsis, acute dyspnoea, acute and severe chronic HF with decreased ejection fraction (HFREF), and HF with preserved ejection fraction are all illnesses that are associated with higher levels of the neurohumoral marker of cardiac biochemical stress known as MR-pro-ADM (HFPEF) [47]. There is evidence that MR-proADM is an early predictor of in-hospital mortality owing to various causes, including respiratory infections, surgical procedures, and cardiovascular illness [48, 49]. 

#### Endothelin-1 (CT-proET-1)

Endothelin-1, a potent vasoconstrictor and potentiator of sympathetic neurohormones generated in blood vessel endothelial cells, is also a sympathetic activation marker. In heart failure, plasma levels of both endothelin-1 and large endothelin-1 are elevated and are linked to pulmonary artery pressure, disease severity, and death [50, 51]. ET-1 and adrenomedullin (ADM) levels in the blood are more significant in people with heart failure than in healthy individuals. It is believed that endothelin has a role in forming pulmonary vasoconstriction, smooth muscle cell proliferation, and remodelling the pulmonary vascular system [52]. In light of this, our team proposed that C-terminal pro-endothelin-1 (CT-proET-1) and mid-regional pro-adrenomedullin (MR-proADM) levels would be higher in heart failure patients compared to controls, and that the degree of this elevation would be related to cardiopulmonary reserve [53, 54]. C-terminal pro-endothelin-1, a newly discovered stable endothelin-1 surrogate marker, has also been proposed as a prognosticator. Increased levels of CT-proET-1, MR-proANP, and MR-proADM were independently linked with the risk of cardiac mortality or heart failure in a recent investigation on patients with stable coronary artery disease after adjusting for clinical cardiovascular risk factors and ejection fraction [55]. 

#### Creatine kinase-MB (CK-MB)

A blood test for creatine kinase has been used to identify myocardial infarction since the 1960s. Because of the discovery of CK-MB, one of three creatine kinase isoenzymes found mainly in the heart, researchers have increased the accuracy of myocardial damage diagnosis and have used it to evaluate acute myocardial infarction (AMI) since the 1970s. CK-MB is one of three isoforms of the creatine kinase enzyme, which is present mainly in the heart but also at minor levels in skeletal muscle [56]. Creatine kinase levels are often elevated in myocardial infarction. As a result, the CK-MB is used to determine whether the elevated creatinine level is due to skeletal or cardiac muscle damage [57]. 

#### Hydroxybutyrate dehydrogenase (HBDH)

The determination of serum hydroxybutyrate dehydrogenase (HBD) activity may be used to diagnose myocardial infarction without the necessity for isoenzyme separation [58, 59]. Raised serum HBDH activity is uncommon in liver disorders, as one would assume [60]. HBDH levels rise in the blood for 8 to 10 h after a heart attack and then rise again in 2–4 days. They may stay high for up to 18 days, which is a considerable period compared to other heart attack indications [60].

#### Heart-fatty acid binding protein (H-FABP)

Heart-fatty acid binding protein (H-FABP) is a low molecular weight cytosolic protein in cardiac tissues that transports fatty acids from the plasma membrane to oxidation sites in mitochondria and peroxisomes, as well as to the endoplasmic reticulum for lipid synthesis [61]. It is primarily found in the myocardium and the brain, kidneys, and skeletal muscle to a lesser level. H-FABP is released into the serum after myocyte rupture very early [62]. H-FABP levels rise as soon as 30 min after myocardial infarction, peak at 6–8 h, and then return to baseline after around 24 h [63]. Aside from that, H-FABP has the potential to be employed as a predictive biomarker of mortality [64].

#### Cardiac Troponin

Cardiac troponin is a calcium-dependent regulator of the heart muscle's contraction mechanism, frequently referred to as a "switch" between contraction and relaxation. For myocardial infarction, cardiac troponin is presently the gold standard biomarker test. Troponin T (TNT), troponin I (TNI), and troponin C (TNC) are the three components of the cardiac troponin complex. Because the amino acid sequences of skeletal and cardiac isoforms of TNT and TNI differ significantly, monoclonal antibody-based immunoassays have been designed to detect cardiac-specific TNT and cardiac-specific TNI.

ACS patients benefit from cardiac troponins because they are sensitive, specific, and offer symptomatic information. As a result, troponin is now the preferred cardiac marker in individuals with ACS. They are also more susceptible to minor degrees of myocardial injury than CK-MB tests. Even though it is the gold standard marker for myocardial infarction, it has minimal usefulness in the diagnosis of reinfarction. A second myocardial infarction that occurs 7 to 10 days after the first may be difficult to identify with cardiac troponin tests alone due to its extended half-life [65].

#### C reactive protein (CRP)

The liver secretes C reactive protein (CRP), one of the acute phase proteins. In reaction to numerous inflammatory cytokines, their content in the blood is quickly growing [66]. More evidence has been gained in recent years concerning the likely involvement of inflammation in developing significant health disorders such as diabetes or heart disease. According to many epidemiological studies, CRP is a powerful independent predictor of future cardiac events, including myocardial infarction, ischemic stroke, peripheral vascular disease without a known heart disease, and sudden cardiac death [67]. This protein is significant because it occurs early in the bloodstream during numerous systemic inflammatory disorders. Lower stability, a lengthy detection period, and cross-reactivity with other blood proteins are some drawbacks in diagnosing this protein [68]. 

#### Tumour necrosis factor (TNF-α)

Tumour necrosis factor (TNF-α) is an inflammatory cytokine that is elevated in chronic HF patients [69]. TNF-α has also been linked to aberrant left atrial dysfunction and advanced left ventricular diastolic and systolic dysfunction disease severity in patients with newly diagnosed HF [70], as well as the use of TNF-α as a predictor of death in advanced HF patients [71].

#### Interleukin-6 (IL-6)

Interleukin-6 (IL-6) is a cytokine that has a role in inflammation, but it also has cardiovascular effects via regulating cardiomyocyte hypertrophy and apoptosis [72]. Cardiac IL-6 expression has been shown to rise in advanced HF, indicating that it may have a role in prognosis [73]. Furthermore, elevated IL-6 levels have been linked to left ventricular dysfunction before HF diagnosis, suggesting that it might be used as a risk marker for the start and progression of HF [74]. This finding suggested that IL-6 may play a role in distinguishing between the decompensated and compensated phases [75].

#### Pentraxin 3 (PTX-3)

In response to an inflammatory stimulus, vascular endothelial cells, smooth muscle cells, macrophages, and neutrophils generate PTX-3, a unique marker of vascular inflammation [76]. In patients with unstable angina pectoris, myocardial infarction, and heart failure, the PTX-3 level has been recommended as a predictive biomarker of poor prognosis [77, 78]. PTX-3, on the other hand, predicts advanced atherosclerosis and is more specific for artery wall inflammation than CRP [79].

#### Pregnancy-associated plasma protein A (PAPPA)

A metalloproteinase called pregnancy-associated plasma protein A (PAPPA) is crucial for the rupture of atherosclerotic plaque [80]. The leading producers are the placental syncytiotrophoblast, fibroblasts, vascular endothelial cells, and vascular smooth muscle cells. It has been linked to plaque progression and instability in atherosclerosis [81].

#### A soluble cluster of differentiation 40 ligands (SCD40l)

The TNF-α family member SCD-40 l is increased on platelets in the intraluminal thrombus. The release of CD40L into the circulation occurs when the inflammatory and coagulant pathways are activated during thrombogenesis, signifying plaque rupture and eventually myocardial infarction [82].

#### Copeptin

The cleavage of pre-PR vasopressin yields copeptin, neurophysin ii, and vasopressin, the latter of which is sometimes referred to as antidiuretic hormone and is crucial for fluid balance and has been associated with the severity of HF [83]. Vasopressin is known to be unstable in circulation, making clinical measures difficult. On the other hand, copeptin is thought to have better stability and is produced in equimolar amounts to vasopressin, making it a more dependable and repeatable option for indirect vasopressin detection [84]. Copeptin may thus be utilized as a diagnostic and prognostic marker for myocardial infarction [85].

#### Growth differentiation factor-15 (GDF-15)

Growth differentiation factor-15 (GDF-15) is a member of the transforming growth factor (TGF) family of cytokines primarily produced by the placenta and implicated in tissue damage inflammatory and apoptotic pathways. GDF-15 levels over a certain threshold have been proven to indicate high-risk individuals with cardiovascular disease [51, 86–88]. Increased GDG-15 levels have been linked to the promotion of protective mechanisms for inhibiting apoptosis, hypertrophy, and adverse remodelling in patients with chronic HF, with increased expression of GDF-15 related to the advancement of protective means for inhibition of apoptosis, hypertrophy, and adverse remodelling [89].

#### F2 Isoprostanes

Arachidonic acid metabolism produces F2 isoprostanes. F2 isoprostanes are released by a few cells, including monocytes, during atherosclerosis. The amount of F2 isoprostane in the urine of individuals with unstable angina is higher in studies. It may also be utilized as a predictor of complications in non-fatal myocardial infarction, the progression of heart failure, and fatality [90].

#### Suppression of tumorigenicity 2 (ST2)

Suppression of tumorigenicity 2 (ST2) is an interleukin (IL)-1 receptor that has been discovered as a target for IL-33, a cytokine produced when the heart is under metabolic stress [90]. ST2 is a soluble protein produced by cardiomyocytes and detectable in the bloodstream. Its levels rise in response to mechanical stress on the heart [91]. Its utility in HF has been recognized as an independent predictor of mortality or the need for transplantation in patients with severe chronic HF [92], as providing prognostic information for patients with acute HF when combined with natriuretic peptides [93], implying that it could be used as a biomarker in conjunction with current clinical testing strategies like BNP and NTPROBNP.

#### Matrix metalloproteinases (MMP) and tissue inhibitors of metalloproteinases (TIMP)

Cardiac extracellular matrix remodelling is an essential mechanism in the development and progression of HF. This remodelling is partly mediated by collagenases, MMP, and TIMP, which degrade collagen and other matrix proteins [94]. Fibrosis and ventricular remodelling may result from an imbalance between MMPS and TIMPS. Excessive collagen crosslinking has been linked to HF hospitalization in hypertensive individuals, according to a new study [95]. George et al. revealed that MMP-2 (but not MMP-3, -9, or TIMP-1) concentrations were an independent predictor of death in patients with HF in the first MMPS investigations [96]. MMP-3 and MMP-9 were univariate indicators of all-cause mortality in patients with HFREF. Only MMP-9 emerged as a poor independent prognosis, according to Buralli et al. [97]. TIMP-1, but not MMP-9, was shown to be of separate and additional value in Frantz et al.'s prediction of all-cause mortality [98, 99]. 

#### Galectin-3

Galectin-3 is implicated in tissue healing, myofibroblast proliferation, and fibrogenesis in the inflammatory pathway after injury and ventricular remodelling. Galectin-3 instillation into the pericardium resulted in a considerable increase in collagen deposition [100]. Galectin-3 is elevated in patients with acute or chronic HF, and univariable analyses are frequently associated with risk, but with adjustment for renal function or other biomarkers, galectin-3 loses its prognostic meaning in many studies [101, 102]. Serial galectin-3 measurements in chronic HF patients may contribute to a single measure, but no known medications may change galectin-3 readings at this time [103].

#### Procalcitonin

Procalcitonin is a pro-peptide of calcitonin typically produced by parafollicular C cells in the thyroid gland [104]. Calcium homeostasis and immunity are regulated by pro-calcitonin/calcitonin axed [105]. There is insufficient proof that serum procalcitonin levels are a reliable marker for chronic HF with predictive value, although serial procalcitonin measurements are advised to differentiate between in-hospital mortality in a variety of diseases associated with pro-inflammatory activation (pneumonia, chronic obstructive pulmonary disease, acute respiratory tract infections, etc.) [106]. Extensive clinical studies are needed to fully clarify the hypothesis and gather further information about the predictive significance of pro-calcitonin in HF patients with worsened symptoms.

#### Comorbidities

Renal dysfunction, hematologic abnormalities, and liver dysfunction are all critical indicators of a poor prognosis in heart failure. While serum creatinine, estimated glomerular filtration rate, and blood urea nitrogen are essential indices of renal function, NT-PROBNP provides additional prognostic information [107]. Their predictive significance is lower at lower levels of impairment, and the commencement of these biomarkers' increase is often delayed following acute renal injury (AKI). Cystatin C and trace protein (BPT) outperformed standard renal indicators in predicting prognosis in HF, owing to its improved capacity to assess renal function at lower degrees of abnormalities [108].

While high levels of NAGL are linked to poor clinical outcomes, this link was lost after controlling for many factors, including NT-PROBNP [109]. Furthermore, it has a reasonable capacity to predict impending AKI (68%sensitivity and 70% specificity) [110]. KIM-1 in the urine and NAG in the blood showed potential, but further research is required. Hematologic abnormalities, including anaemia, iron deficiency, and increased red blood cell distribution width (RDW), are common in HF patients and play an essential role in predicting prognosis, independent of LVEF [111]. It is uncertain if correcting hematologic abnormalities can enhance clinical outcomes in HF patients.

#### Myeloperoxidase (MPO)

Myeloperoxidase (MPO) is an enzyme secreted into an extracellular fluid by polymorphonuclear neutrophils and macrophages' azurophilic granules during inflammation. A valuable risk marker and diagnostic tool in acute coronary syndrome and chest discomfort are myeloperoxidase, which is implicated in oxidative stress and inflammation [112]. High concentrations of MPO, irrespective of traditional CVD risk factors, have been demonstrated to enhance CVD risk in epidemiological investigations [113, 114]. MPO is implicated in all phases of endotheliosis, from endothelial dysfunction to plaque rupture, according to in vitro studies, and it is involved in the initiation of CVD and acute cardiovascular events [115]. Since both myoglobin and haemoglobin have peroxidase activity and the substrate for myeloperoxidase (MPO) and general peroxidase is the same, detecting them is problematic [116].

#### Fibrinogen

In 1836, fibrinogen was identified and characterized as the first clotting agent [117]. Cells participating in the atherogenic process may produce cytokines, which trigger an acute phase response that raises plasma fibrinogen levels [118]. Fibrinogen is a significant acute phase protein that creates the substrate for thrombin and constitutes the last stage in the coagulation cascade [119]. According to research, fibrinogen is a significant and independent cardiovascular risk factor related to established risk factors and genetic variants. Despite unsolved difficulties requiring definitive solutions, fibrinogen has emerged as an essential new predictor of cardiac risk [120] since we may detect low fibrinogen levels in individuals with severe hepatic illness [121].

#### Trimethylamine n-oxide (TMAO)

Trimethylamine n-oxide (TMAO) is a dietary metabolite linked to red meat consumption. Liver enzymes produce TMAO from the gaseous precursor trimethylamine (TMA), which is created when gut bacteria break down these nutrients [122]. In all diets, red meat has the highest concentration of l-carnitine, increasing the quantity of l-carnitine accessible for TMAO production. While a recent study has proven that high levels of TMAO in the blood are linked to an elevated risk of heart disease and death, it shows that TMAO may be used as a biomarker for both heart and kidney illness [123].

#### Cystatin C

Most nucleated cells regularly produce and release cystatin C, a tiny protease inhibitor. It is a well-described blood marker of renal failure in clinical practice. In addition, because of decreased renal function and the relationship between cardiac risk and illnesses, cystatin C is being studied as a cardiac marker [124].

#### Myoglobin

Myoglobin is an oxygen-binding protein that forms a compound with iron molecules in skeletal and cardiac muscle tissues. One hour after myocardial infarction, it is released into circulation. Within 2 to 4 h after the commencement of myocardial damage, it steadily rises, peaks in 8–12 h, and recovers to normal in 24–36 h. Due to substantial levels of myoglobin in skeletal muscles, myoglobin is an early indication of acute myocardial infarction but lacks cardiac specificity. Myoglobin should not be used alone to confirm a diagnosis of acute myocardial infarction; instead, it should be combined with other heart-specific markers like TNI or TNT to boost diagnostic value [125].

#### Ischemia-modified albumin (IMA)

When serum albumin from ischemic cardiac tissues comes into touch with IMA, it becomes a new sign of ischemia. The albumin cobalt binding test, based on the inability of IMA to bind cobalt, has been developed and commercialized as the first commercially accessible FDA-approved diagnostic of myocardial ischemia with a 30-min laboratory turnaround time [126]. IMA may distinguish ischemic and non-ischemic patients. Because IMA is elevated in illnesses other than chest pain, its ineffectiveness calls into doubt its specificity. Even while IMA assessment remains the sole clinical biomarker for myocardial ischemia, the process of IMA generation and the precise unit being evaluated are unknown [127].

#### Endothelial dysfunction (noninvasive method)

Endothelial cells are a monolayer of active cells lining the inside blood and lymphatic arteries. It acts as an active paracrine, endocrine, and autocrine organ to maintain vascular homeostasis [128]. It controls oxidative stress, as well as both local and systemic inflammation. According to current research on atherosclerosis and the basic mechanisms involved, harmful changes in endothelial physiology and biology, also known as endothelial dysfunction, may be a critical early step in the development of atherosclerosis and may also signal lesion progression and the occurrence of complications [129, 130]. Endothelial dysfunction is an independent predictor of poor cardiovascular outcomes and may have more predictive power than established risk variables alone in predicting future cardiovascular events [131].

#### Apoptosis antigen-1 (APO1/FAS)

FAS, also known as apoptosis antigen-1 (APO1), is a TNF family member that regulates apoptosis. FAS is present in the thymus, liver, ovary, lung, and heart, among other organs [132, 133]. FAS is increased in cardiomyocytes due to apoptotic cell death caused by hypoxia and overstretching [134]. A soluble version of FAS (SFAS) missing the transmembrane domain was also discovered in human serum (133), and its levels are expected to be higher in individuals with cardiovascular disease. Individuals with severe CHF had considerably greater SFAS levels in their blood than patients with moderate CHF. Furthermore, stepwise multivariate analysis revealed that individuals with high SFAS and BNP and a poor ejection fraction had a substantially greater mortality rate than those with low SFAS levels, indicating that plasma SFAS is a helpful prognostic marker in CHF aetiology.

#### Neutrophil gelatinase-associated lipocalin (NGAL)

Neutrophil gelatinase-associated lipocalin (NGAL) is a lipocalin protein having a polypeptide chain of 178-a.a and a molecular mass of 25 KDa expressed by neutrophils and different epithelial cells express [135, 136]. NGAL expression is elevated under acute phase protein [137, 138]. NAGL seems to be one of the first kidney indicators of nephrotoxic and ischemia damage in animal models, and it is shown to be high in the urine and blood of people shortly after acute injury (AKI) [138]. Compared to control participants, chronic HF patients had more significant amounts of NGAL in both blood and urine [139]. NGAL seems to function as a biomarker in the case of acute HF, according to research by Damman et al. As a result, further study into the function of NGAL in acute and chronic heart failure might be beneficial [140].

#### Uric acid (UA)

In humans, uric acid (UA) is the final product of purine metabolism. The inactivation of uricase and the subsequent rise in UA levels are hypothesized to have offered evolutionary benefits by guarding against oxidative damage [141]. Increased serum UA, even below the clinical threshold for hyperuricemia, has been linked to the development of CVD through increasing oxidative stress, inducing endothelial dysfunction, and boosting inflammation [142]. Recent research has shown an independent link between UA and cardiovascular mortality [143, 144]. However, the evidence supporting the outcomes is still inconclusive. Numerous epidemiological investigations, including prospective, retrospective, cross-sectional, and meta-analysis studies, have failed to find an independent link between UA and CVD [145, 146].

#### Neuregulin-1 (NRG-1)

Endothelial cells emit neuregulin-1 (NRG-1), which binds to a family of ERBB receptors on neighbouring cardiac myocytes to enhance cell survival, growth, and maintenance [147]. The NRG-1 gene has been linked to more than 15 distinct protein products. The most prevalent NRG-1 protein in the cardiac system is NRG-1. The paracrine impact of the NRG-1 ligand is mediated by the ERBB (ERBB2, ERBB3, ERBB4) tyrosine kinase receptor family. NRG-1 is activated by various cardiovascular stressors, including oxidative stress, ischemia, and exercise [148]. As a result of NRG-1/ERBB4 paracrine signalling in the heart, this system may be engaged in cardiac adaptation to different types of physiologic stress. NRG-1 levels were associated with more advanced stages of HF and a poorer outcome in HF patients with CAD [149]. Increased blood levels of NRG, like NT-PROBNP, may be an insufficient physiologic response to cardiovascular injury, and exogenous treatment of NRG may enhance cardiovascular function. These data support the theory that NRG-1 in the myocardium is activated in response to ischemia. NRG-1's potential as a CVD biomarker needs further investigation.

#### Human serum albumin (HAS)

Albumin is a significant protein in human plasma made by liver cells. Albumin has a variety of activities, including controlling fluid filtration and adsorption across capillary walls and transporting various chemicals in the bloodstream. Albumin concentrations are often evaluated in blood or urine and may be used as indicators of dehydration, malnutrition, liver or kidney illness, and cardiovascular disease [150].

#### Serum amyloid A (SAA)

The serum amyloid A (SAA) protein belongs to a group of apolipoproteins that are usually linked to high-density lipoproteins (HDL). When interleukin-6 (IL-6) is stimulated in response to infection, inflammation, injury, or stress, the acute phase SAA proteins SAA1 and SAA2 are released into the blood. Binding phase proteins are being examined extensively as possible indicators for predicting the progression of cardiovascular illnesses, and SAA has shown to be a promising candidate for a cardiac biomarker [151, 152].

#### Retinol-binding protein 4 (RBP4)

Retinol-binding protein 4 (RBP4) is a member of the lipocalin protein family that serves as a carrier protein for vitamin A in serum. RBP4 has been shown to have a critical role in insulin resistance. Several studies have recently revealed that RBP4 levels in the blood may be linked to cardiovascular disease and metabolic syndrome [153, 154].

#### Soluble lectin-like oxidized LDL receptor (SLOX-1)

The extracellular domain of LOX-1 is proteolytically cleaved to form the soluble lectin-like oxidized LDL (SLOX-1) receptor. LOX-1 is a transmembrane protein present on the cell surface of endothelial cells and smooth muscle cells, among other places. SLOX-1 has been suggested as a biomarker for plaque rupture in a few studies, and it may have clinical usefulness in detecting atherosclerosis-related disorders [155, 156].

#### Adiponectin (ADN)

Adipokines are a kind of protein hormone abundant in the human body. It is a crucial lipid and glucose metabolism regulator predominantly expressed by adipocytes. According to research, ADN is an insulin sensitizing hormone with anti-diabetic, anti-inflammatory, and anti-atherogenic characteristics. ADN levels in the blood have been linked to various lifestyle disorders, including atherosclerosis and heart failure [157, 158].

#### S100 protein

S100 proteins have a molecular weight of 10 to 12 KDa and are acidic calcium-binding proteins. In humans, there are over 20 distinct members of this family. These proteins seem to be involved in various biological activities, including cell proliferation and differentiation, as well as the inflammatory response. The involvement of several family members (S100B and S100A) in heart illness has been investigated [159].

### Lipid derived biomarkers

#### Triglyceride-to-high-density lipoprotein cholesterol ratio

Triglyceride (TG) to high-density lipoprotein cholesterol ratio (HDLC) and total cholesterol (TC)-to-HDLC ratio, as well as a low ankle branchial pressure index (ABPI), are vital biomarkers for CVD [160]. Although triglyceride levels may be seen in most routine lipid panels, they can also be utilized to predict CVD risk by combining them with other lipid profile indicators [160]. A triglyceride-to-HDL cholesterol ratio of more than 3.5 seems to be linked to an elevated risk of cardiovascular disease. Apolipoprotein B (APO-B), lipoprotein A (LPA), high-density lipoprotein cholesterol (HDL-C), and low-density lipoprotein cholesterol (LDL-C) elevations are reported during HF. Important biochemical markers include a considerable drop in apolipoprotein A1 (APOA1), APOA1/APOB ratio, and PON1 activity/HDLC ratio [161]. People with type 2 diabetes mellitus (T2DM) who have higher levels of LDL, triglycerides, and total cholesterol (hyperlipidemia) have a greater risk of coronary artery disease (168). Total cholesterol, LDL-C, APOB, LP(A), and the APOA1/APOB proportions are also excellent biomarkers for predicting CVDs in people [162].

#### Low-density lipoprotein cholesterol

A high plasma concentration of low-density lipoprotein cholesterol (LDLC) is a crucial risk factor for atherosclerotic cardiovascular disease (ACD). Furthermore, a rapid increase in LDLC plasma content suggests a significant risk of cardiovascular disease and necessitates faster therapeutic intervention [163]. Similarly, autosomal dominant hypercholesterolemia (ADH) is characterized by high LDL cholesterol, which causes early morbidity and death due to atherosclerotic cardiovascular disease (ASCVD). Similarly, familial combination hyperlipidemia (FCHL) is characterized by elevated total cholesterol, triglycerides, or LDL cholesterol and a significant risk of ASCVD [164]. LRP1 (low-density lipoprotein receptor-related protein 1) is a large endocytic and signalling receptor mainly found in the liver, where it regulates blood coagulation factor VIII plasma levels by controlling its absorption and subsequent degradation. Similarly, tiny dense LDL cholesterol is a new risk factor for cardiovascular disease [165]. 

#### Lipoprotein-associated phospholipase A2 (LP-PLA2)

Lipoprotein-associated phospholipase A2 (LP-PLA2) is an enzyme that functions in blood vessel inflammation and is thought to contribute to atherosclerosis. This test determines how much LP-PLA2 is present in the blood. Recent research has shown that LP-PLA2 is a standalone risk factor for cardiovascular disease (CVD), including coronary heart disease (CHD) and ischemic stroke [166]. The sensitivity C-reactive protein (HS-CRP) test is linked to systemic inflammation, and higher levels are linked to an increased risk of cardiovascular disease. The LP-PLA2 test is linked to vascular inflammation and is considered to raise the risk of cardiac events, such as heart attack or stroke, at high levels. In persons with diabetes, a high level of LP-PLA2 is linked to vascular problems such as kidney damage [167]. Although it is a risk factor for heart disease, decreasing LP-PLA2 activity with medication is not advised for people with stable CHD [168].

#### Oxylipin

Oxylipins are bioactive lipid metabolites formed when polyunsaturated fatty acids are oxidized by the cyclooxygenase (COX), lipoxygenase, and cytochrome p450 pathways. They have a role in immunological, inflammatory, and vascular activities [169]. Abnormal oxylipin signalling has also been associated with CVD risk factors such as hypertension, hyperlipidemia, thrombosis, haemostasis, and diabetes. Oxylipins are a new age of risk indicators in numerous illnesses, including cardiovascular disease, and various oxylipins have a role in CVD development, albeit the mechanism is yet unknown [170]. 

### Pre-disease biological marker

#### Hypertension (HTN)

Hypertension is a disease of the modern world. Although it seldom causes symptoms, it affects 16% to 37% of the world's population and is a significant risk factor for coronary artery disease, stroke, HF, and atrial fibrillation (AF) [171]. The available data support the claim that blood pressure (BP) monitoring is a substantial predictor of long-term risk and that it is better than clinic blood pressure measurement for this purpose. As a result, BP measurement combines higher precision with cheap cost and ease of application. According to American and European standards, all patients with known or suspected hypertension should have their BP checked and controlled at home [172, 173].

### Nucleic acid-derived biomarkers

#### MicroRNAs (miRNAs)

MicroRNAs (miRNAs) are noncoding RNA molecules with a short length. They act via the seed region, a six- to an eight-nucleotide sequence that binds to messenger ribonucleic acid (mRNA) known as miRNA targets [174]. They primarily inhibit gene expression by downregulating translation at the post-expression stage, using two fundamental pathways: translational repression and mRNA degradation. The most reliable approach for quantitatively comparing miRNA expression levels is a real-time quantitative polymerase chain reaction, which has been the cornerstone of miRNA quantification. Lipid metabolism, glucose homeostasis, vascular integrity, and endothelial cell function are only a few pathways influenced by miRNA control [175, 176]. Earlier studies revealed that a miRNA panel's combined utility enhanced the predictive value of classic Framingham risk models, but no single miRNA imparted a clinically relevant reduction in acute MI risk [177].

### Genome-based biomarkers

#### Soluble ST2

Over the last decade, more research has revealed that soluble ST2 is substantially more significant in people with heart failure and can aid with risk assessment for both acute and chronic heart failure. The US Food and Drug Administration recently approved a commercial-grade soluble ST2 assay (Presage, Critical Diagnostics, San Diego, CA) for use in chronic heart failure prognosis [49, 178, 179].

#### Blood gene expression in CAD

The Corus CAD (CardioDx, Palo Alto, CA) assay is now commercially available for this gene expression test. By standard protein biomarkers, the test is confined to patients with chest pain who do not have diabetes, chronic inflammatory illnesses, increased leukocyte levels, or acute coronary syndromes. The clinical efficacy of a transcriptome profile Corus CAD must be extensively validated against these current noninvasive standards and investigated in various groups [180, 181].

#### DEFA1/DEFA3

DEFA1/DEFA3 were the most strongly linked with CHD development among eight possible markers in a cross-sectional study of CHD prediction biomarkers in non-familial hyperlipidemia patients, and show promise for further application as inflammatory markers to synergistically predict the risk of CHD development in Thai hyperlipidemia patients [182, 183].

## Conclusions

Over the last decade, various efforts have been undertaken to discover meaningful biomarkers for cardiovascular disease risk management. The search for the most beneficial and ideal cardiovascular indicators will likely go on forever. Many ancient and innovative substances will be studied as single markers and in multi-marker techniques in the future years. We explored the notion that an ideal cardiovascular biomarker may simultaneously provide clinicians with a range of information concerning predictive, diagnostic, and prognostic implications, in addition to satisfying the conventional biomarker designation requirements. Based on the gathered data, a few circulating molecules seem to be of interest in this regard since they can provide all the clinical data described above. However, further research is needed before an effective platform for biomarker analysis that may replace conventional CVD diagnosis in clinical practice can be provided. Innovative targets are expected to be discovered due to the expansion of public biomarker databases, advancements in the sensitivity and selectivity of analytical techniques, and the development and routine use of innovative platforms with proven potential.

## Data Availability

Not applicable.
